# Chemical Oxidative Polymerization of 2-Aminothiazole in Aqueous Solution: Synthesis, Characterization and Kinetics Study

**DOI:** 10.3390/polym8110407

**Published:** 2016-11-23

**Authors:** Hua Zou, Lu Wang, Xia Wang, Pengfei Lv, Yaozu Liao

**Affiliations:** 1 School of Materials Science and Engineering, University of Shanghai for Science and Technology, Shanghai 200093, China; hua.zou@usst.edu.cn (H.Z.); wanglu1390636@163.com (L.W.); feiniao416@163.com (P.L.); 2 State Key Laboratory for Modification of Chemical Fibers and Polymer Materials, College of Materials Science and Engineering, Donghua University, Shanghai 201620, China

**Keywords:** poly(2-aminothiazole), chemical oxidative polymerization, yield, thermal stability, kinetics

## Abstract

The chemical oxidative polymerization of 2-aminothiazole (AT) was studied in aqueous solution using copper chloride (CuCl_2_) as an oxidant. The effect of varying the reaction temperature, reaction time and oxidant/monomer molar ratio on the polymer yield was investigated. The resulting poly(2-aminothiazole)s (PATs) were characterized by FTIR, ^1^H NMR, UV-vis, gel permeation chromatography, scanning electron microscopy, thermogravimetric analysis and four-point probe electrical conductivity measurements. Compared with a previous study, PATs with higher yield (81%) and better thermal stability could be synthesized. The chemical oxidative polymerization kinetics of AT were studied for the first time. The orders of the polymerization reaction with respect to monomer concentration and oxidant concentration were found to be 1.14 and 0.97, respectively, and the apparent activation energy of the polymerization reaction was determined to be 21.57 kJ/mol.

## 1. Introduction

Conducting polymers have attracted considerable attention for several decades, among which the most widely studied examples are the heterocyclic polymers, such as polyaniline, polypyrrole and polythiophene [1]. 2-Aminothiazole (AT) is a heterocyclic amine combining the chemical structures of aniline, pyrrole and thiophene. Owing to the existence of sulfur- and nitrogen-rich subgroups, AT and its derivatives demonstrated excellent adsorption properties to heavy metal ions [2,3,4,5,6,7,8]. For example, AT-modified silica gels were able to adsorb and preconcentrate Ni(II), Cu(II), Cd(II), Zn(II) and Pb(II) [7]; also, AT-functionalized polyacrylonitrile and polystyrene exhibited an excellent selectivity for adsorption of Hg(II) and Au(III) ions in aqueous solution [8]. Due to the high sulfur and nitrogen atom molar ratio (30%) in AT molecules, which is much higher than that in aniline (7%) and pyrrole (10%), it is expected that the adsorption capacity of AT would be considerably enhanced after polymerization [9]. 

Similar to other heterocyclic conducting polymers, both electrochemical and chemical oxidative syntheses of poly(2-aminothiazole) (PAT) have been studied. Considerable effort has been devoted to electrochemical polymerization of AT [10,11,12,13,14,15]. However, there are only a few studies on synthesis of PAT by chemical oxidative polymerization, which has an obvious advantage in production of polymers on a large scale. The chemical oxidative polymerization of AT has been conducted using different oxidant/solvent systems, including sodium hypochloride/acetic acid aqueous solution [16,17], CuCl_2_·6H_2_O/1,4-dioxane [18], benzoyl peroxide/1,4-dioxane [19], and anhydrous FeCl_3_/nitromethane (CH_3_NO_2_) at a low oxidant/monomer molar ratio (as low as 0.01) [20]. 

**Scheme 1:**
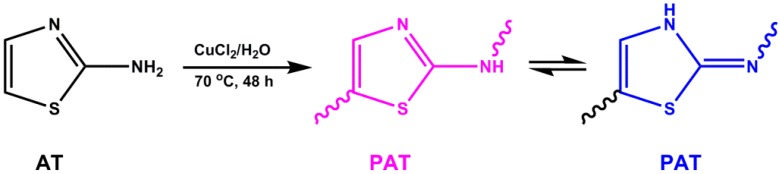
Synthetic route to poly(2-aminothiazole) PAT with two tautomeric forms.

In a recent work [21], we described the preparation of PAT by chemical oxidative polymerization using a low amount of copper chloride (CuCl_2_·2H_2_O) as an oxidant in aqueous solution (see Scheme 1), which is known as a cheap, non-toxic, and environment-friendly solvent. It is noteworthy that the polymerization was conducted without any acid being added. However, that paper focused on the adsorption properties of PAT for Hg(II) in aqueous solution. In the present work, a systematic investigation of the chemical oxidative polymerization of AT in aqueous solution is reported. The optimal conditions for synthesis were determined by examination of the effect of varying reaction temperature, reaction time, and oxidant/monomer molar ratio on the yield of PAT. The structure, morphology, thermal stability and conductivity of the polymers were investigated. On the other hand, the kinetics of chemical polymerization of pyrrole and aniline have been well studied with a wide range of experimental techniques in the literature [22,23,24,25,26,27,28]. However, as far as we are aware, no study has been undertaken on the kinetics of the chemical polymerization of AT. In this paper, the chemical oxidative polymerization kinetics of AT were studied for the first time. This work lays the foundation for our ongoing research on the preparation of colloidal dispersions of PAT.

## 2. Materials and Methods

### 2.1. Materials

AT (97%, Sigma-Aldrich, St. Louis, MO, USA) and CuCl_2_·2H_2_O (99%, Sinopharm Chemical Reagent, Shanghai, China) were used as received. All water used in these experiments was deionized (>18 MΩ) using a Milli-Q water purification system (Millipore Corporation, Billerica, MA, USA).

### 2.2. Synthesis of PAT

A typical synthesis of PAT was conducted as follows: 4.0 g (40 mmol) of AT monomer and 100 mL of H_2_O were charged into a 250 mL three-neck flask equipped with a magnetic stirrer, a thermometer, a Graham condenser and a heating mantle. The solution was then stirred and heated to 70 °C, followed by dropwise addition of 20 mL of aqueous solution containing 1.38 g (8 mmol, oxidant/monomer molar ratio of 0.2) of CuCl_2_·2H_2_O. The color of the solution changed from pale yellow to brown-black once the oxidant solution was added. The reaction was allowed to proceed for various reaction times. After the reaction, the polymer was washed thoroughly three times with deionized water. All the products were dried in a vacuum oven to constant weight. Polymer yields of the polymerization reaction were calculated based on the method normally used for chemical synthetic conducting polymers by dividing the mass of the original PAT by that of the AT used.

### 2.3. Kinetics Studies

The kinetics of the polymerization were studied by monitoring the PAT concentration in the early stage of the polymerization using a parallel reactor [29]. A series of experiments were conducted at 70 °C at different time intervals (10–110 min) to establish the dependence of the initial polymerization rate (*R*_i_) of AT on monomer concentration (AT) and oxidant concentration (CuCl_2_). The polymerization reaction order with respect to monomer concentration was determined by conducting the polymerization reaction at different monomer concentrations (AT) (0.1–0.4 mol/L) and a fixed oxidant concentration of 4 mmol/L. The polymerization reaction order with respect to oxidant concentration was determined by conducting the polymerization reaction at different oxidant concentrations (CuCl_2_) (0.1–0.4 mol/L) and a fixed monomer concentration of 0.4 mol/L. The thermodynamic parameters were determined by conducting the polymerizations at 50, 60, 70 and 80 °C at a fixed monomer concentration (0.4 mol/L) and a fixed oxidant concentration (0.4 mol/L) at different time intervals (10–110 min).

### 2.4. Characterization

FTIR spectra were recorded on a PerkinElmer Spectrum 100 FTIR spectrometer (PerkinElmer, Waltham, MA, USA) in KBr. ^1^H NMR spectra were taken using a BrukerAvance 400 spectrometer (Bruker, Billerica, MA, USA). UV-vis absorption spectra were recorded using a Lambda 750 UV-vis spectrophotometer (PerkinElmer, Waltham, MA, USA) in dimethyl sulfoxide solution. Molecular weight data were obtained by gel permeation chromatograms (GPC, Agilent Technologies, Santa Clara, CA, USA) with an Agilent 1100 system using dimethylformamide as an eluent. The morphology and diameter of the polymer particles were evidenced by scanning electron microscopy (SEM, FEI, Amsterdam, The Netherlands) on a Quanta FEG 450 microscope. Samples were dried and gold-coated prior to examination. Thermogravimetric analysis (TGA) was carried out with a PerkinElmer Pyris 1 TGA apparatus (PerkinElmer, Waltham, MA, USA) in N_2_ at a heating rate of 10 °C/min from room temperature to 600 °C. Electrical conductivities were measured under ambient condition using a standard four-probe method on compressed pellets with a probe station CascadeM150 connected to the Keithley 4200 semiconductor analyzer (Keithley Instruments Inc., Cleveland, OH, USA).

## 3. Results and Discussion

### 3.1. Optimization of the Polymerization Conditions

**Figure 1:**
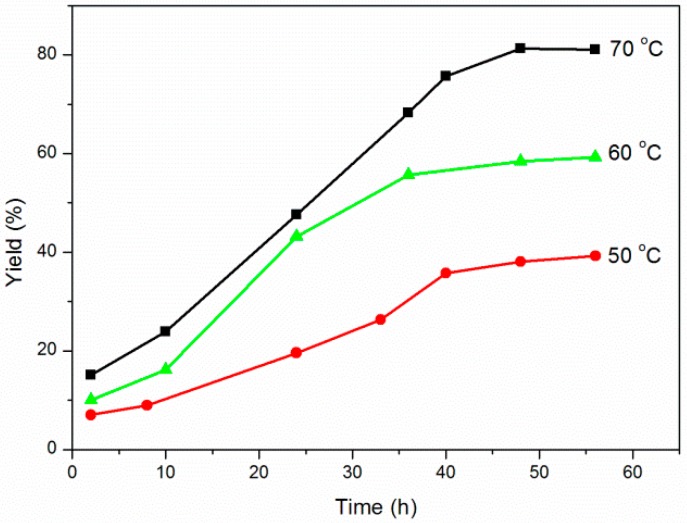
Effects of polymerization temperature and time on the yield of PAT at synthesized at a fixed oxidant/monomer molar ratio of 0.2.

The effects of reaction temperature, time and oxidant/monomer molar ratio on the yield of PAT were studied to optimize the synthesis. Figure 1 presents the effect of polymerization temperature and time on the polymer yields at a fixed oxidant/monomer molar ratio of 0.2. The polymer yield increased substantially with increasing polymerization temperature. Meanwhile, the polymer yield increased with increasing polymerization time from 1 h to approximately 40 h. However, with further increase of the polymerization time to 56 h, the polymer yield increased slightly. Therefore, 70 °C and 48 h were selected as the optimal polymerization temperature and time, respectively. This also suggests that the polymerization probably proceeds via a step-by-step mechanism. 

**Figure 2:**
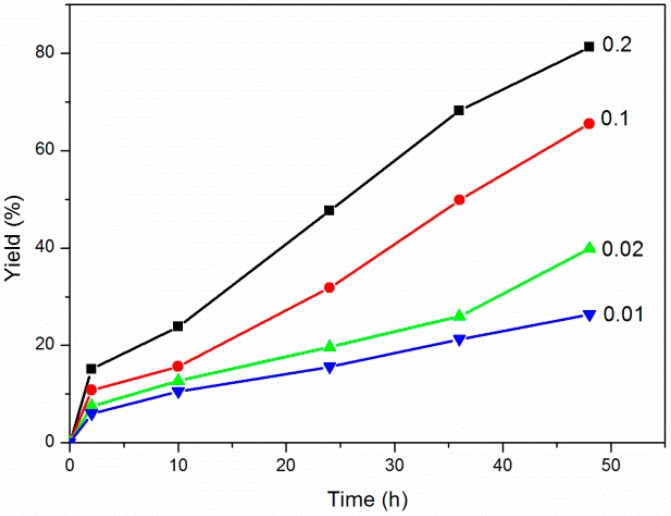
Effects of oxidant/monomer molar ratio and polymerization time on the yield of PAT synthesized at 70 °C.

Figure 2 shows the effects of oxidant/monomer molar ratio and polymerization time on the yield of PAT synthesized at 70 °C. As expected, the polymer yield was improved with increasing oxidant/monomer molar ratio. This is reasonable because a higher oxidant/monomer molar ratio can generate more radical species, which can accelerate the polymerization reaction and improve the polymer yield. A very high yield of PAT (~81%) was obtained when the polymerization time was 48 h and the oxidant/monomer molar ratio was 0.2, whereas the yield was 78% with FeCl_3_ as an oxidant in nitromethane under the same conditions [20].

### 3.2. Structure and Morphology

The chemical structure of PAT was characterized with FTIR and ^1^H NMR spectroscopies (see Figures S1 and S2, respectively). The results are similar to our previous studies [20,21], suggesting that the polymerization was initiated by the oxidation of −NH_2_.

**Figure 3:**
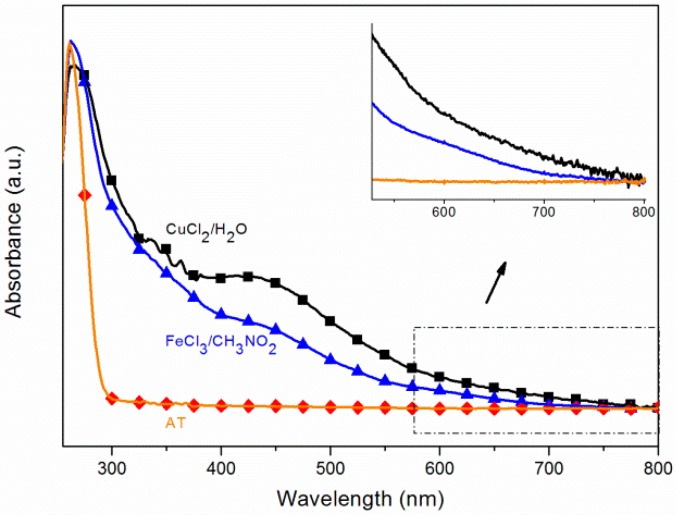
UV-vis spectra of 2-aminothiazole (AT) and PATs synthesized with different oxidant/solvent systems at the same conditions (70 °C, 48 h and oxidant/monomer molar ratio of 0.2).

Figure 3 shows the UV-vis spectra of AT and PATs synthesized with different oxidant/solvent systems but under identical other conditions (70 °C, 48 h and oxidant/monomer molar ratio of 0.2). The spectrum of AT showed a peak at 267 nm belonging to the π → π* transition of the thiazole ring. Compared with that of the AT monomer, it is obvious that the spectra of the PATs are considerably red shifted, which is a clear evidence of the formation of conjugation after polymerization [20]. The spectrum of PAT synthesized with CuCl_2_/H_2_O not only has a peak at ~267 nm, but also has a broad absorption band at around 450 nm with a long tail extending to 750–800 nm, which could be attributed to the π → π* transition of the conjugated polymer. The onset wavelengths were red shifted with decreasing oxidant/monomer molar ratio from 0.2 to 0.01 (see Figure S3), probably due to the enhanced conjugation length and molecular weight. 

Compared with that synthesized with FeCl_3_/CH_3_NO_2_, the spectrum of PAT synthesized with CuCl_2_/H_2_O shows a weaker absorption band and a shorter tail, indicating the PAT probably has a longer conjugation length and higher molecular weight. To confirm this possible correlation, the molecular weight of PAT was determined by GPC. The number-average molecular weight (*M*_n_) and weight-average molecular weight (*M*_w_) were 5.90 × 10^3^ and 8.12 × 10^3^, respectively, higher than those synthesized with FeCl_3_/CH_3_NO_2_ under the same conditions (*M*_n_ = 4.48 × 10^3^ and *M*_w_ = 7.32 × 10^3^) [20], respectively. This can be explained by the fact that CuCl_2_ is a milder oxidant than FeCl_3_, resulting in a lower rate of polymerization and higher molecular weights.

**Figure 4:**
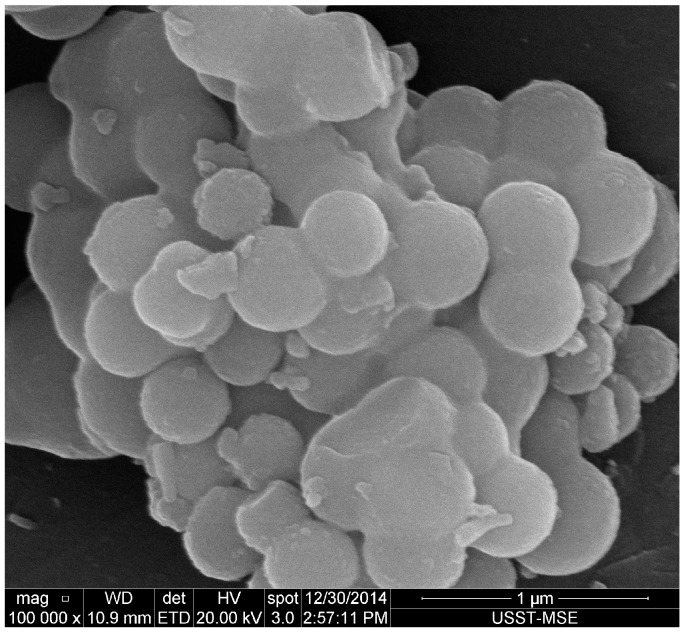
A representative SEM image depicting the granular morphology of PAT powder (prepared at 70 °C, 48 h and oxidant/monomer molar ratio of 0.2).

The morphology of the PAT powder was examined by SEM. As shown in Figure 4, the particles had a granular morphology with an average size of approximately 400 nm. This is the most common morphology observed for conducting polymers, as the typical conditions used for the preparation of conducting polymer by chemical oxidative polymerization involve a high concentration of nucleates that are produced during short induction period [30]. Due to the limited solubility of nucleates in water, they separate from aqueous medium. The random aggregation of nucleates followed by polymer-chain growth thus gives rise to the globular morphology [31]. This concept of morphology formation was proposed for polyaniline but it also seems to be applicable also to related polymers such as polypyrrole and PAT. For example, similar morphology was observed for polypyrrole nanoparticles prepared by oxidative H_2_O_2_ initiated polymerization [32]. It should also be mentioned that the mean size of the granules was much larger than that prepared with FeCl_3_/CH_3_NO_2_, indicating that the morphology of the PAT was substantially affected by the oxidant/solvent system.

### 3.3. Thermal Stability

**Figure 5:**
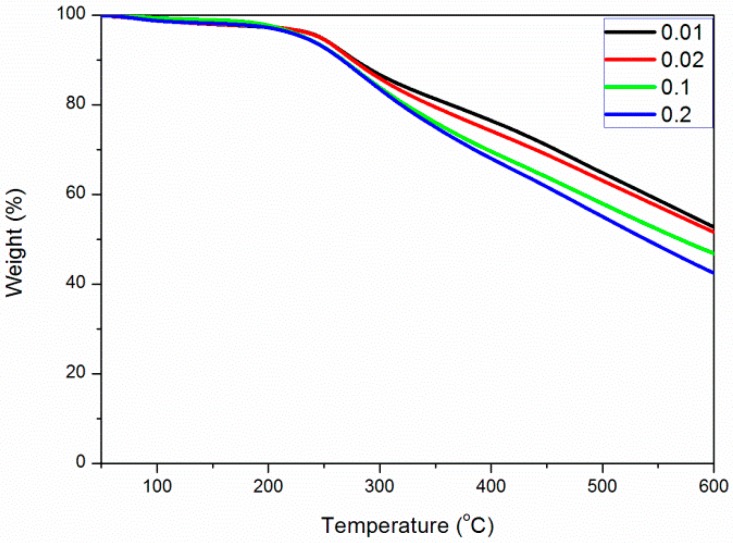
TGA curves of PATs synthesized at various oxidant/monomer molar ratios (70 °C, 48 h).

The TGA curves of PATs synthesized at various oxidant/monomer molar ratios are presented in Figure 5. It is shown that the thermal stability of PATs increased with decreasing oxidant/ monomer molar ratio, indicating that PATs with longer conjugation length and higher molecular weight were formed at a lower oxidant/monomer molar ratio. This is supported by our GPC results, which showed that the *M*_n_ values were 5.9 × 10^3^, 26.9 × 10^3^ and 199 × 10^3^, at the oxidant/monomer molar ratios of 0.2, 0.1, and 0.02, respectively. For oxidant/monomer molar ratio of 0.01, the GPC data could not be obtained due to the poor solubility of the resulting polymer.

We further compared the initial decomposition temperature of PATs prepared in this work and those prepared with FeCl_3_/CH_3_NO_2_ in our previous study [20]. The results are shown in Table 1. Obviously, PATs prepared with CuCl_2_/H_2_O showed much better thermal stability than those prepared with FeCl_3_/CH_3_NO_2_. This could also be ascribed to their longer conjugation length and higher molecular weight, which is in good agreement with our UV-vis and GPC study.

### 3.4. Electrical Conductivity

Table 2 shows the electrical conductivities of PATs before and after doping with I_2_. The original PATs showed poor conductive properties (≤1.0 × 10^−13^ S/cm). These conductivity values were in very good agreement with previous studies [16,20]. However, the electrical conductivities of PATs could be improved by around 9 orders of magnitude up to 2.1 × 10^−4^ S/cm upon I_2_ doping for 24 h. 

### 3.5. Kinetics of Polymerization

We examined how the rate of polymerization of AT was affected by the monomer concentration, the oxidant concentration and the temperature in the early stages of the polymerization. 

#### 3.5.1. Effect of Monomer Concentration

**Figure 6:**
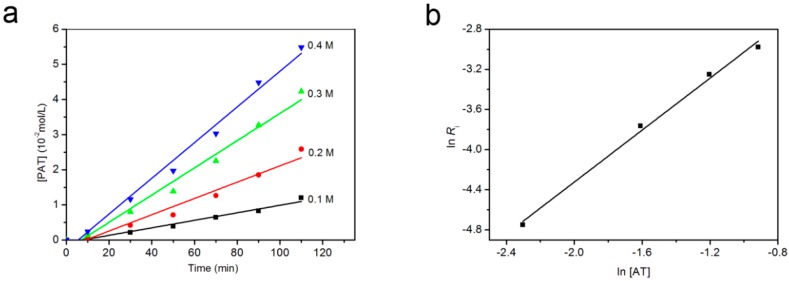
(**a**) Effect of monomer concentration on the concentration of PAT at different time intervals; (**b**) plot of ln *R*_i_ versus ln [AT].

Chemical oxidative polymerization of AT was carried out at 70 °C at different monomer concentrations (AT) (0.1–0.4 M) at different time intervals (10–110 min). As shown in Figure 6a, polymer concentration (PAT) was found to vary linearly with time. The initial polymerization rate (*R*_i_) at corresponding monomer concentration could be obtained from the slope according to the following equation:

(1) $$R_{i}=\frac{W_{\mathrm{PAT}}}{V\times M_{\mathrm{AT}}\times t}=\frac{\left[\mathrm{PAT}\right]}{M_{\mathrm{AT}}\times t}$$

where *W*_PAT_ is the weight of obtained polymer, *V* is the volume of the reaction mixture in litre, *M*_AT_ is the molecular weight of the monomer, and *t* is the time. 

Based on the *R*_i_ values at different monomer concentrations (AT) obtained from Figure 6a, the relationship between ln *R*_i_ and ln (AT) was established, see Figure 6b. The slope of this linear relationship was found to be 1.14 ± 0.03, indicating that the order of the polymerization reaction with respect to the monomer concentration was 1.14 ± 0.03. Thus the initial rate of the polymerization reaction increases with increasing monomer concentration.

#### 3.5.2. Effect of the Oxidant Concentration

**Figure 7:**
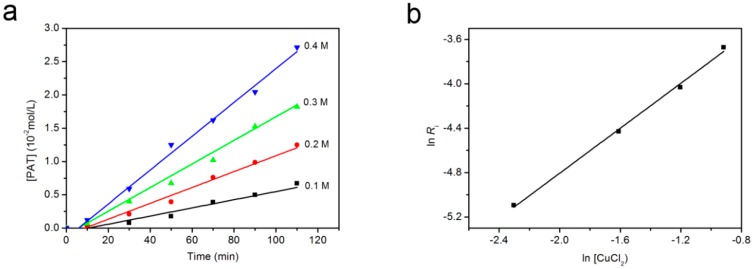
(**a**) Effect of oxidant concentration on the concentration of PAT at different time intervals; (**b**) the plot of ln *R*_i_ versus ln (CuCl_2_).

Similarly, the chemical oxidative polymerization of AT was carried out at 70 °C at different oxidant concentrations (CuCl_2_) (0.1–0.4 mol/L) at different time intervals (10–110 min). As shown in Figure 7a, the plot of polymer concentration (PAT) versus *t* at each (CuCl_2_) was also found to be linear, and each initial polymerization rate (*R*_i_) was obtained from the slope of the line. Then the relationship between ln *R*_i_ and ln (CuCl_2_) was established, see Figure 7b. The slope of this linear relationship was found to be 0.97 ± 0.05, indicating that the order of the polymerization reaction with respect to the oxidant concentration was 0.97 ± 0.05. Thus the initial rate of polymerization reaction increases with increasing oxidant concentration.

#### 3.5.3. Calculation of the Thermodynamic Parameters

**Figure 8:**
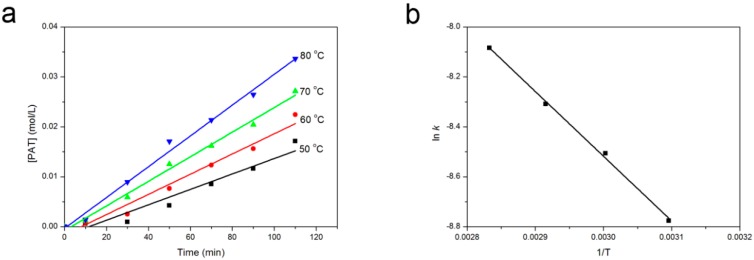
(**a**) Effect of polymerization temperature on the concentration of PAT at different time intervals; (**b**) the plot of ln *k* versus 1/*T*.

To study the effect of polymerization temperature, chemical oxidative polymerizations of AT were further carried out at 50, 60, 70, and 80 °C at a fixed monomer concentration (0.4 mol/L) and a fixed oxidant concentration (0.4 mol/L) at different time intervals (10–110 min). The [PAT]-time curves were shown in Figure 8a, and *R*_i_ values at different temperatures were obtained from the slope of the lines. Meanwhile, *R*_i_ could be expressed by the following reaction rate equation:

*R*_i_ = *k*(AT)^1.14^(CuCl_2_)^0.97^ (2)

where *k* is the reaction rate constant. According to this equation, the values of *k* at 50, 60, 70 and 80 °C were calculated to be 1.07 × 10^−3^, 1.40 × 10^−3^, 1.70 × 10^−3^ and 2.13 × 10^−3^, respectively.

The apparent activation energy (*E*_a_) of the polymerization of AT was calculated according to the following Arrhenius law:

(3) $$\ln k=\frac{-E_{a}}{RT}+\ln A$$

where *E*_a_ is the activation energy, *R* is the universal gas constant 8.314 J mol^−1^·K^−1^, *T* is the reaction temperature (K), and *A* is a constant. The Arrhenius plot of the natural logarithm of *k* versus the reciprocal of temperature (1/*T*) for the polymerization of AT is shown in Figure 8b. This plot was linear, indicating that the reaction obeyed the Arrhenius law. The activation energy (*E*_a_) calculated from the slope was 21.57 ± 0.72 kJ/mol. This value is much lower than the activation energy of the initiation step of chemical polymerization of pyrrole in water using the oxidant FeCl_3_ (79.485 kJ/mol) [28], and the activation energy of chemical polymerization of poly-*o*-phenylenediamine in aqueous hydrochloric acid solution using K_2_Cr_2_O_7_ as oxidizing agent (63.658 kJ/mol) [29], indicating that the polymerization reaction in this study can be readily achieved.

The enthalpy (Δ*H*) and entropy (Δ*S*) of activation were calculated by the following Eyring equation [29]:

(4) $$k=(\frac{RT}{Nh})e^{\Delta S/R}.e^{-\Delta H/RT}$$

where *N* is the Avogadro’s number, *R* is the universal gas constant, and *h* is Planck’s constant. The following equation was further obtained by simple mathematical operations:

(5) $$\ln(\frac{k}{T})=\ln(\frac{R}{Nh})+\frac{\Delta S}{R}+\frac{-\Delta H}{RT}$$

**Figure 9:**
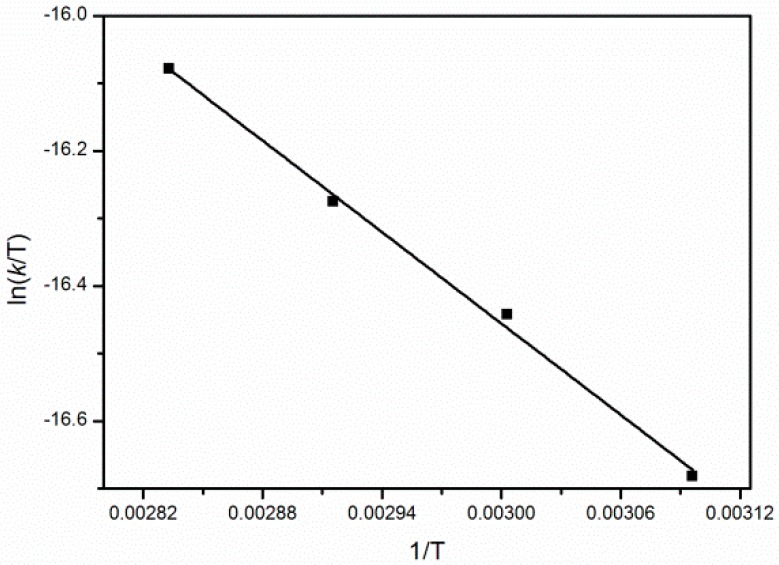
The plot of of In (*k*/*T*) versus 1/*T*.

The relationship between ln(k/T) versus 1/T is shown in Figure 9, giving a linear relationship with (−ΔH)/R as the slope and (ln (R/Nh) + ΔS/R) as the intercept. The values of ΔH and ΔS were then calculated to be 18.76 ± 0.73 kJ·mol^−1^ and 244.32 ± 2.16 J·mol^−1^·K^−1^, respectively. The positive value of ΔH indicates the reaction is endothermic [29], while the positive value of the ΔS means that the entropy of the transition state is more disordered than the entropy of the reactants [28].

## 4. Conclusions

In this work, the chemical oxidative polymerization of AT by CuCl_2_ in aqueous solution was studied systematically. When the polymerization time was 48 h and the oxidant/monomer molar ratio was 0.2, a very high yield of PAT (~81%) was achieved at 70 °C. In comparison with that prepared using FeCl_3_ as an oxidant in nitromethane under the same conditions, PAT synthesized with CuCl_2_/H_2_O had a higher yield, longer conjugation length and higher molecular weight. The polymer had a granular morphology, which is common for conducting polymers. The thermal stability of PATs increased with decreasing oxidant/monomer molar ratio and the electrical conductivities of PATs could be remarkably improved up to 2.1 × 10^−4^ S/cm upon I_2_ doping for 24 h. 

The chemical oxidative polymerization kinetics of AT in the early stage of the polymerization was studied for the first time. The initial rate of polymerization reaction increases with increasing monomer and oxidant concentrations. The exponents of monomer and oxidant were found to be 1.14 and 0.97, respectively, and the apparent activation energy was determined to be 21.57 kJ/mol. This indicates that the polymerization reaction can be readily achieved. Both the enthalpy and entropy of activation were positive, revealing that the reaction is endothermic and the entropy of the transition state is more disordered than the entropy of the reactants. 

## Figures and Tables

**Table 1:** Comparison of the initial decomposition temperature of PATs prepared with CuCl_2_/H_2_O and FeCl_3_/CH_3_NO_2_ (70 °C, 48 h).

Oxidant/Monomer molar ratio	CuCl_2_/H_2_O (°C)	FeCl_3_/CH_3_NO_2_ (°C) [20]
0.2	220	135
0.1	224	165
0.02	236	183
0.01	240	210

**Table 2:** Effect of oxidant/monomer molar ratio and doping time on electrical conductivity of PAT (70 °C, 48 h).

Oxidant/Monomer molar ratio	Original (S/cm)	12 h (S/cm)	24 h (S/cm)
0.2	1.0 × 10^−14^	1.0 × 10^−7^	1.3 × 10^−5^
0.1	5.0 × 10^−14^	3.6 × 10^−7^	3.9 × 10^−5^
0.02	3.0 × 10^−14^	9.0 × 10^−7^	1.7 × 10^−4^
0.01	1.0 × 10^−13^	4.0 × 10^−6^	2.1 × 10^−4^

## Supplementary Materials

The following are available online at www.mdpi.com/2073-4360/8/11/407/s1. Figure S1: FT-IR spectra of AT and PAT; Figure S2: PAT with two tautomeric forms and ^1^H NMR spectra of AT and PAT; Figure S3: UV-vis spectra of AT and PAT synthesized with various oxidant/monomer molar ratio.
