# Supplementary material for: Chemical Oxidative Polymerization of 2-Aminothiazole in Aqueous Solution: Synthesis, Characterization and Kinetics Study
Source: Polymers (Basel). 2016 Nov 23;8(11):407. doi: 10.3390/polym8110407 (PMC6432296; doi:10.3390/polym8110407)
Supplement: Supplementary file 1 [file polymers-08-00407-s001.pdf]

Supplementary Material: Chemical Oxidative Polymerization of 2-Aminothiazole in Aqueous Solution: Synthesis, Characterization and Kinetics Study

Hua Zou, Lu Wang, Xia Wang, Pengfei Lv and Yaozu Liao

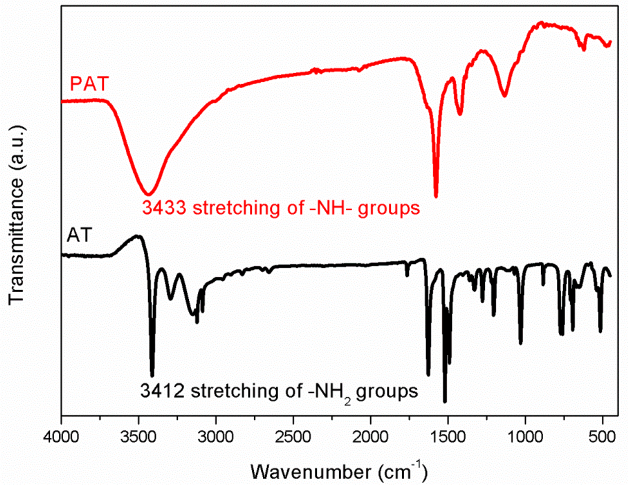

Figure S1. FT-IR spectra of AT and PAT (prepared at 70 °C, 48 h and oxidant/monomer molar ratio of 0.2).

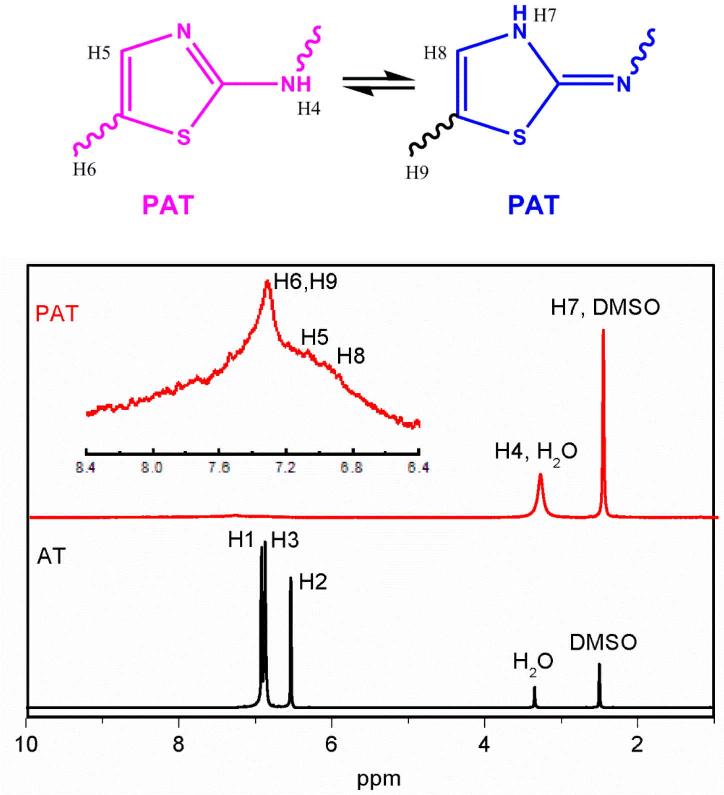

Figure S2. PAT with two tautomeric forms (**top**) and ^{1}H NMR spectra (**bottom**) of AT and PAT (prepared at 70 °C, 48 h and oxidant/monomer molar ratio of 0.2).

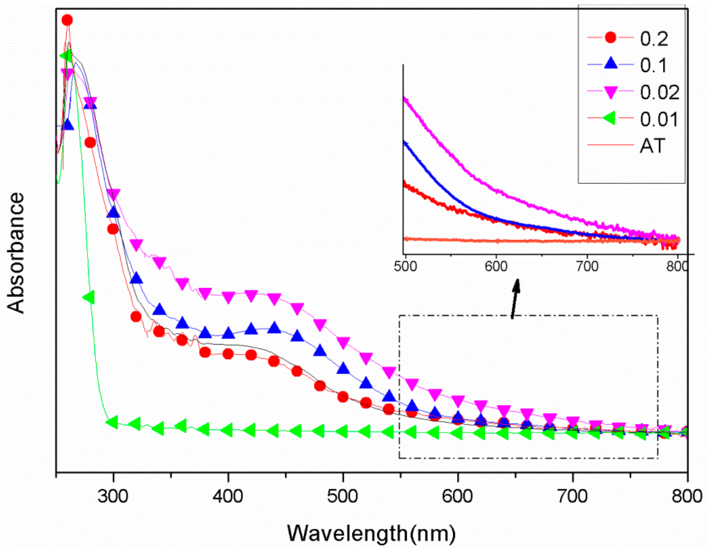

Figure S3. UV-vis spectra of AT and PAT synthesized with various oxidant/monomer molar ratio (70 °C, 48 h).
